# Metabolic, Hormonal and Body Condition Changes in Melatonin-Implanted Dairy Rams and Flock Pregnancy Rate During the Mating Season: A Longitudinal Field Study

**DOI:** 10.3390/ani16142196

**Published:** 2026-07-15

**Authors:** Francesca D. Sotgiu, Claudia Caporali, Antonio Spezzigu, Matteo Sini, Chiara C. Costantino, Andrea Mattu, Valeria Pasciu, Christopher Odey, Francesca Mossa, Fiammetta Berlinguer

**Affiliations:** 1 Department of Veterinary Medicine, University of Sassari, 07100 Sassari, Sardinia, Italy or claudia.caporali@outlook.it (C.C.); matteosini888@gmail.com (M.S.); c.costantino1@studenti.uniss.it (C.C.C.); andreamattu99@gmail.com (A.M.); vpasciu@uniss.it (V.P.); c.odey@phd.uniss.it (C.O.); fmossa@uniss.it (F.M.); berling@uniss.it (F.B.); 2 EmbryoSardegna, Tecnologia, Riproduzione e Fertilità, 07034 Perfugas, Sardinia, Italy; antospezzigu@gmail.com

**Keywords:** dairy rams, reproductive performance, metabolic status, faecal thyroid hormone metabolites, faecal corticosteroid metabolites, mating season

## Abstract

Reproductive activity imposes significant physiological demands on rams. This study evaluated metabolic and hormonal fluctuations in Sarda rams under semi-extensive management conditions, together with flock reproductive outcomes. Fourteen rams were isolated from ewes, subjected to nutritional flushing, and treated with melatonin implants (3 × 18 mg) before joining the flock. From June to December, body condition score (BCS), non-esterified fatty acids (NEFA), urea, triglycerides, cholesterol, testosterone, faecal thyroid hormone metabolites (FTMs), and faecal corticosteroid metabolites (FCMs) were measured every 45 days. Ewes’ pregnancy rates (PRs) and conception dates were determined by ultrasound scanning to estimate flock reproductive performance. BCS declined (*p* < 0.05) from June (3.11 ± 0.06) to November (2.80 ± 0.06). In November, NEFA, cholesterol and FCMs peaked (*p* < 0.05), whereas triglycerides and urea reached the lowest levels (*p* < 0.05). FTMs peaked in June and November (*p* < 0.05). Testosterone concentrations were three-fold higher in June than the rest of the mating season (*p* < 0.05), while overall PR was stable. Despite metabolic and endocrine changes in rams, overall flock reproductive performance remained stable. The final stage of mating season may represent a strategic period for targeted management strategies to sustain welfare and long-term performance.

## Simple Summary

Traditional breeding management of dairy sheep farms in Mediterranean countries relies almost exclusively on natural mating. This requires rams to withstand a long mating season (MS), usually extending from late spring to late autumn. Despite their vital role in flock productivity, ram management is typically prioritised only before the MS, with limited welfare monitoring during this period. To assess how rams cope with the reproductive effort, this study evaluated fluctuations in selected metabolites (non-esterified fatty acids (NEFA), urea, triglycerides, cholesterol), hormones (testosterone, faecal thyroid—FTMs and corticosteroid metabolites—FCMs), and body condition score (BCS) in Sarda rams (*n* = 14) raised under a semi-extensive system during the MS. Flock reproductive performance was assessed by measuring pregnancy rates (PR) via transrectal ultrasound scanning and retrospective estimation of conception dates. Results showed a decline in rams’ BCS and a metabolic shift in November in response to increased metabolic demand, while overall PR remained stable. Results indicate that Sarda rams mobilised body reserves to meet mating season energetic demands, during which pregnancy rates remained stable. These findings suggest the need for rams’ targeted management during the mating season to support welfare, recovery, and long-term efficiency.

## 1. Introduction

Dairy sheep production represents a major economic, environmental, and socio-cultural component of the livestock sector in Mediterranean countries [1]. Under semi-extensive traditional management systems, breeding is predominantly based on natural mating, typically with one lambing per year, with the mating season starting in late spring for adult ewes, resulting in lambing between October and December, whereas nulliparous ewe lambs are typically mated in late summer–early autumn and lamb [2] between January and March. Induced mating protocols, including the ‘ram effect’, exogenous hormonal treatments, and dietary supplementation [3,4,5,6,7,8], are often applied to stimulate both male and female reproductive activity [2,8]. Reproductive activity is particularly demanding for rams, as they undergo an energetically taxing and prolonged mating season, typically extending from late April/early May to early November, during which they must mate with both adult ewes and ewe lambs. During the reproductive period, rams are exposed to a range of fluctuating environmental factors (air temperature, humidity, photoperiod, and/or pasture availability) and social conditions, mostly linked to the shift in flock dynamics throughout the season. This dynamic is driven by the introduction of ewe lambs to the initial adult flock, which temporarily increases the ewe-to-ram ratio. This is followed by the progressive removal of pregnant ewes, ultimately leading to a scarce availability of females that can intensify male–male interactions toward the end of the season. In this context, the intense physical exertion during mating may impose a substantial energetic burden, potentially affecting metabolic homeostasis and the neuroendocrine pathways regulating reproduction [9,10,11,12]. It is well established that environmental stressors, such as extreme temperatures and housing conditions, alongside social stressors, such as mating competition among males [13,14], can significantly influence the rams’ physiological status and reproductive performance [9,15,16].

Consequently, these combined challenges can temporarily disrupt the animals’ homeostatic balance, triggering an array of adaptive responses [17] that can impact male welfare and potentially influence overall flock reproductive outcomes.

General health, libido, and mating ability are typically assessed in rams shortly before the start of the breeding season [18,19]. However, the comprehensiveness of these evaluations is often limited by economic and time constraints. Furthermore, while several long-term studies to date have explored variations in different hormonal and/or metabolic markers in rams, mainly in relation to semen quality or testicular size [20,21,22,23,24], limited knowledge exists regarding the fluctuations of these markers in response to the aforementioned metabolic, environmental, and social challenges that occur during the breeding season [25]. We hypothesised that in rams supplemented with exogenous melatonin, the metabolic and endocrine milieu may vary across the mating season, reflecting the changing physiological demands of reproductive activity.

We further hypothesised that assessing the metabolic and hormonal profiles in rams, together with pregnancy and conception rates in ewes, could provide a descriptive overview of physiological and reproductive dynamics during the mating season and help identify critical periods in which targeted management interventions may be beneficial.

Building on these premises, this study aimed to characterise fluctuations in metabolic and hormonal biomarkers in rams during the mating season under field farming conditions. Metabolic biomarkers, including circulating non-esterified fatty acids (NEFA), cholesterol, triglycerides, and urea, were assessed as indicators of energy balance [6]. Hormonal biomarkers, including concentrations of circulating testosterone, faecal thyroid metabolites (FTMs), and faecal corticosteroid metabolites (FCMs), were selected to evaluate the activity of the hypothalamic–pituitary–gonadal, thyroid, and adrenal axes, which regulate reproductive activity and physiological adaptation to metabolic and environmental challenges [10]. Body condition score (BCS) was included as an integrative indicator of long-term energy reserves [26]. Finally, pregnancy and conception rates were assessed as flock-level reproductive outcomes to contextualise the observed endocrine and metabolic fluctuations within the overall reproductive dynamics of the flock and to identify potential periods requiring targeted management interventions.

## 2. Materials and Methods

### 2.1. Ethical Statement

The animal study protocol was approved by the Local Committee for Animal Welfare at the University of Sassari (protocol code 5266, date of approval 23 January 2025). All experimental procedures performed in the present study followed the DPR 27/1/1992 (“Animal Protection Regulations of Italy”) and European Community Regulation 86/609.

### 2.2. Animals and Reproductive Management

The study was conducted on a commercial farm located in northern Sardinia, Italy (40°46′14.5″ N 8°24′10.2″ E). Fourteen fertile Sarda rams, aged between 2 and 4 years, were enrolled.

From March to mid-May (approximately two months before the onset of the mating season), all rams were housed together in an indoor pen, in complete visual, olfactory, and auditory isolation from the ewes. During this period, the daily diet consisted of 2 kg/head of a commercial pelleted feed divided into two rations, with grass hay and water provided ad libitum. In early April, as part of the farm’s routine reproductive management practice, each ram received three subcutaneous slow-release melatonin implants (18 mg MELOVINE^®^, Ceva Salute Animale SPA, Milan, Italy) to advance the onset of the breeding season [27].

In mid-May, the rams were introduced to a flock of 784 adult ewes managed in a single paddock. The animals were fed a unifeed diet supplemented with 2 kg/head of a commercial pellet (16–17% CP), hay, and water ad libitum, and had access to pasture for 3 to 6 h per day depending on the season. Subsequently, in late June, 218 ewe lambs were added to the flock. The rams remained with the females until mid-November, when they were finally separated. Ewes did not receive any exogenous hormonal or melatonin treatment; the advancement of the breeding season was achieved exclusively through the ‘ram effect’ [2,27], as a result of the introduction of sexually active rams into the flock.

Ewe pregnancy rates were monitored from June to December through five consecutive transrectal ultrasound examinations, conducted every 45 days. Scanning was conducted using a SonoScape S8 scanner (SonoScape Europe Ltd., Rome, Italy) equipped with a 10 MHz rigid linear transducer. Pregnancy was confirmed by the presence of a corpus luteum, an enlarged uterine horn filled with anechoic fluid, and a detectable embryonic heartbeat [6]. Concurrently, foetal age was estimated by measuring the crown-rump length, thoracic diameter, and biparietal diameter [28].

**Figure 1:**
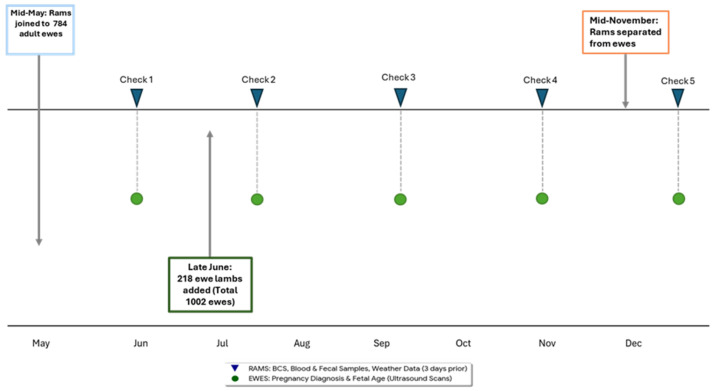
Timeline of the experimental design. Sampling points (triangles) indicate BCS assessment, blood and faecal sampling, and recording air temperatures from 3 days prior to each check. Circles represent ultrasound assessments for pregnancy diagnosis and foetal age estimation. Rectangles indicate management phases.

The timeline of the experimental design is shown in Figure 1.

### 2.3. Determination of Body Condition Score, Endocrine and Metabolic Status of Rams

From June to December, all rams underwent five visits at approximately 45-day intervals. Average, maximum, and minimum air temperatures (T_mean; T_max; T_min) for the three days preceding each visit were retrieved from a nearby weather station (40°38′22.74″ N 8°17′33.12″ E) and processed in the R environment (R 4.4.0 version), using *GSODR* package (5.0.1 version). This time window was chosen because faecal metabolites provide an integrated measure of hormonal activity, reflecting physiological responses to environmental conditions over the preceding 2–3 days [29,30]. During these visits, Body Condition Score (BCS) was assessed by the same operator on a scale from 0 (extremely emaciated) to 5 (obese), according to the method described by Russel et al. [31]. Blood and faecal samples were concurrently collected from all rams. Fasting blood samples were drawn from the jugular vein into 9.0 mL vacuum collection tubes spray-coated with K2EDTA (Vacutainer Systems Europe; Becton Dickinson, Le Pont-de-Claix, France). Immediately after collection, samples were chilled to 4 °C. Blood was then centrifuged at 1500× *g* for 20 min at 4 °C; plasma was separated and stored in vials at −20 °C until assayed. Faecal samples were collected directly from the rectum using a gloved hand, immediately placed on ice, and subsequently stored at −20 °C. All plasma and faecal samples were analysed in duplicate.

#### 2.3.1. Biochemical Analyses

Circulating concentrations of non-esterified fatty acids (NEFA) (DiaSyS, Diagnostic Systems, Holzheim, Germany), total cholesterol, urea, and triglycerides (Hagen Diagnostica Srl, Firenze, Italy) were measured using the enzyme endpoint method at 550 nm, 510 nm, 340 nm, and 546 nm, respectively. Normal Serum I and Abnormal Serum II (FUJIFILM Wako Chemicals Europe, GmbH, Neuss-Uedesheim, Germany) were used as quality control for NEFA analysis. Clinical Chemistry Level 2 and Level 3 controls (Randox Laboratories Ltd., Crumlin, UK) were used for total cholesterol, urea and triglycerides. Calibration was performed using the specific standards provided by the manufacturer for each analyte: 1 mmol/L for NEFA, 50 mg/dL for urea, 200 mg/dL for total cholesterol, and 2.28 mmol/L for triglycerides. The intra-assay and inter-assay CV values and detection limits were: 1.07%, 0.98% and 0.01 mmol/L (for NEFA), 1.7%, 1.6% and 4.9 mg/dL (for urea), 0.95%, 1.24% and 5 mg/dL (for total cholesterol), and 0.99%, 1.05% and 3 mg/dL (for triglycerides), respectively.

#### 2.3.2. Hormonal Analyses

Testosterone circulating levels were quantified with a commercial kit (Ria Testosterone IM1087-01, Beckman Coulter, Prague, Czech Republic). Blood plasma samples were thawed and extracted according to the following procedure: 200 μL of sample was added with 2 mL of ethyl ether, shaken vigorously and frozen at −20° C. Subsequently, the organic phase was separated from the aqueous phase and evaporated at 37° C, then re-dissolved in 200 μL of extraction buffer. Assay sensitivity was 0.05 ng/mL, and the intra- and interassay CVs were 11.6 and 13.5%, respectively.

Faecal samples were processed to determine faecal thyroid hormone metabolites (FTMs) and faecal corticosteroid metabolites (FCMs). All samples were thawed at room temperature and manually mixed using a spatula to ensure uniformity before extraction. The ensuing extraction procedure and analytical methods for determining FTMs and FCMs followed different steps.

##### Fecal Thyroid Hormone Metabolite Analysis

From each homogenised sample, 0.2 g of faeces were lyophilised in 15 mL plastic tubes. Then, FTMs were extracted following a protocol previously described by Pasciu et al. [32]. FTM levels were determined using an ELISA kit supplied by DiaMetra Srl (REF DK0044, PerkinElmer company, High Wycombe, UK).

##### Fecal Corticosteroid Metabolite Analysis

Faecal samples (0.5 g) were homogenised with 80% aqueous methanol according to a procedure described by Palme, followed by centrifugation. The resulting supernatant was then analysed using a specific EIA for faecal glucocorticoid metabolites [33].

All 96-well flat-bottom ELISA plates were read at 450 nm using a microplate reader (FLUOstar Omega; BMG Labtech, Ortenberg, Germany) suitable for standard SBS-format multiwell plates and equipped with BMG Labtech software Mars Data Analysis V5.03.

### 2.4. Statistical Analysis

Statistical analysis was performed with R version 4.4.0 (R Core Team 2024) using the lme4 package (version 2.0-1).

Differences in the serially measured biological variables were analysed using linear mixed-effects models, including time and age as a fixed effect and animal as a random effect to account for repeated measures.

The general form of the model was:

*Y_ij_* = *μ* + *T_i_* + *A_ij_* + *b_j_* + *ε_ij_*

where *Y_ij_* represents the response variable, *μ* is the overall mean, *T_i_* is the fixed effect of time of measurement, *A_ij_* is the fixed effect of age, *b_j_* is the random effect of the *j*-th ram, and *ε_ij_* is the residual error term.

A compound symmetry (CS) type covariance structure was used to model within animal correlation across repeated measures.

Model assumptions were assessed through graphical inspection of residuals (QQ plots and residuals vs. fitted plots). Variables not satisfying normality and homoscedasticity assumptions (triglycerides, testosterone, FTMs, and FCMs), were log-transformed prior to analysis. For these variables, results are presented as back-transformed estimated marginal means on the original scale. Pairwise comparisons were performed using Tukey-adjusted tests based on estimated marginal means. Statistical significance was set at *p* < 0.05. Results are expressed as Estimated Marginal mean ± standard error of the mean (SEM) unless otherwise stated. Relationships among variables were assessed using Pearson’s correlation coefficient.

Reproductive outcomes were indirectly estimated by analysing the findings obtained from the five consecutive reproductive ultrasound examinations of the ewes. At each flock visit, pregnancy rate (PR = number of ewes diagnosed as pregnant/number of ewes exposed to the rams) and the reproductive load (number of available females per ram) were calculated. To ensure this load accurately reflected the actual mating opportunities, the number of available ewes was dynamically updated by excluding individuals that had been diagnosed as pregnant in the previous visit. In the event of an abortion, the affected ewes were immediately removed from the flock and temporarily considered unavailable; they re-entered the mating pool after a 21-day interval, which corresponded to the period required for post-abortion treatment.

Because PR is inherently influenced by the number of ewes a ram must serve, the varying reproductive load across checks made the observed PRs not directly comparable over time. To account for these demographic fluctuations, PRs were standardised using a generalised linear binomial model. By applying the average ewe-to-ram ratio calculated across all checks, a standard load of 27 ewes per ram was established, allowing for a fair comparison of reproductive outcomes throughout the entire experimental period.

A simulation-based power analysis was performed using the final linear mixed-effects models to evaluate the adequacy of the sample size. The analysis was conducted using the simr package in R (version 1.0.9, 1000 simulations). Although the study included 14 rams, each animal was repeatedly sampled across five experimental periods, resulting in a longitudinal dataset comprising approximately 70 observations. The estimated statistical power for detecting the effect of sampling period ranged from 87.8% to 100% across all investigated variables, indicating that the study was adequately powered to detect biologically relevant differences.

## 3. Results

### 3.1. Temporal Dynamics of Reproductive Load and Pregnancy Rates

**Figure 2:**
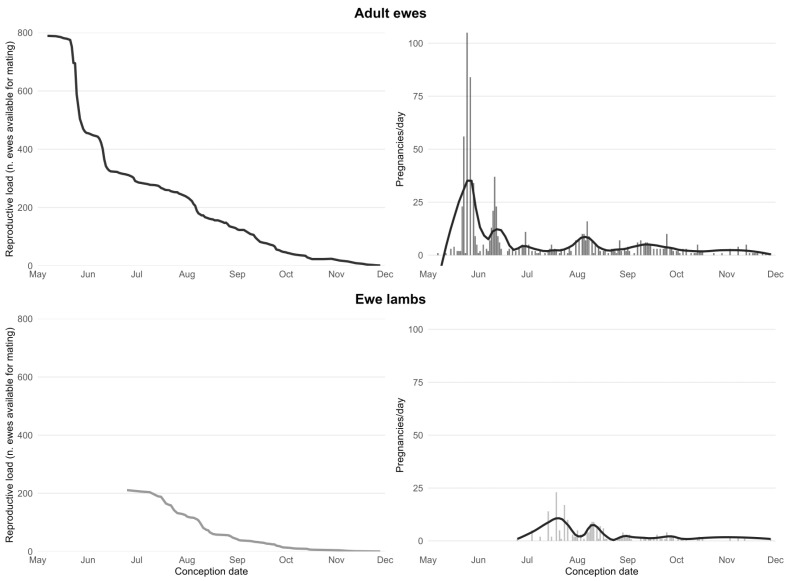
Daily number of conceptions and the number of females available for mating across the mating season in adult ewes (*n* = 784, **upper panels**) and ewe lambs (*n* = 218, **lower panels**). **Left panels** show the temporal decline in the number of adult and ewe lambs available for mating, reproductive load, while **right panels** display the daily number of pregnancies (bars), with a LOESS-smoothed trend (black line) highlighting temporal patterns in conceptions.

The daily distribution of pregnancies (Figure 2) revealed distinct patterns between adult ewes and ewe lambs. In ewes, conceptions were concentrated at the onset of the mating season, followed by a progressive decline. In contrast, ewe lambs exhibited a more dispersed distribution of conceptions over time, with a slight peak between July and August. This trend resulted in a sharp initial reduction in the reproductive load, which then decreased more gradually over time.

**Figure 3:**
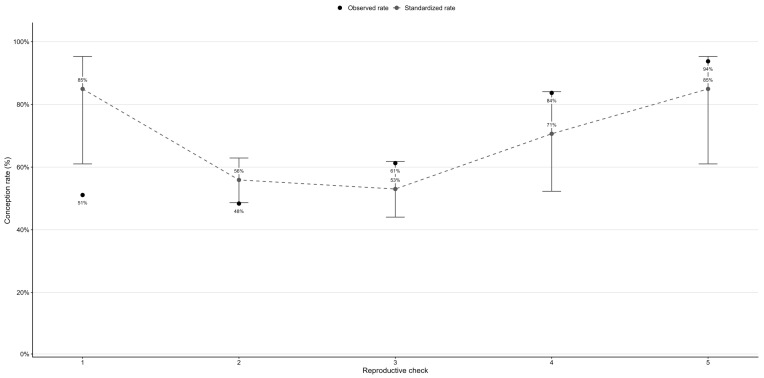
Flock conception rate at the standard load (27 ewes/ram) across 5 distinct reproductive ultrasound checks spaced approximately 45 days apart, performed throughout the mating season. Grey dots represent the standardised conception rates; black dots represent the observed conception rates.

The evaluation of reproductive outcomes conducted under a constant standardised load of 27 ewes per ram revealed a distinct temporal trend compared to the non-standardised data (Figure 3). In this standardised scenario, the pregnancy rate (PR) peaked at the first check (85%), followed by a decline during the second (56%) and third checks (53%). Subsequently, the PR recovered at the fourth (71%) and fifth checks (85%), returning to levels comparable to the onset of the mating season.

The observed PR values at the first and second checks showed negative deviations of −34% and −8% from the standardised rates, respectively. In contrast, the subsequent three checks yielded positive deviations of +8%, +13%, and +9%.

### 3.2. Biochemical, Hormonal, and Body Condition Changes During the Mating Season

The mean external temperature was 19.98 °C in June, 23.45 °C in July, 24.13 °C in September, 17.34 °C in November, and 14.53 °C in December. The differences between maximum and minimum mean temperatures were 9.45 °C in June, 8.75 °C in July, 15.30 °C in September, 8.18 °C in November, and 6.0 °C in December. The mean BCS during the mating season was 2.94, ranging from 3.11 to 2.77. BCS varied over time (*p* < 0.001) with higher values (*p* < 0.05) observed in June and July (3.11 ± 0.06 and 3.12 ± 0.06, respectively) compared to September, November and December (2.77 ± 0.06; 2.80 ± 0.06, and 2.86 ± 0.06, respectively).

No significant effect of age was detected for any of the variables analysed (Supplementary Table S1). Circulating NEFA and cholesterol varied during the mating season (*p* < 0.001), with mean concentrations of 0.167 mmol/L and 47.38 mg/dL, and ranges of 0.09–0.36 mmol/L and 43.5–54.8 mg/dL, respectively. NEFA concentrations were higher in November (0.36 ± 0.02 mmol/L) than in June (0.16 ± 0.02 mmol/L), July (0.11 ± 0.02 mmol/L), September (0.11 ± 0.02 mmol/L) and December (0.09 ± 0.02 mmol/L; *p* < 0.05). Similarly, cholesterol was higher in November (54.8 ± 2.15 mg/dL) compared to July (46.6 ± 2.15 mg/dL), September (43.6 ± 2.15 mg/dL) and December (43.5 ± 2.15 mg/dL; *p* < 0.05).

Urea concentrations varied over time (*p* < 0.001), with a mean of 20.9 mg/dL (range: 13.2–29.1 mg/dL), and were higher in June (24.0 ± 1.42), July (23.6 ± 1.42) and December (29.1 ± 1.42 mg/dL) than in September (14.4 ± 1.42) and November (13.2 ± 1.42 mg/dL; *p* < 0.05). Triglycerides also varied across checks (*p* = 0.001), with a mean of 24.6 mg/dL (range: 16.9–27.8 mg/dL), and were lower in November (16.9 ± 2.15 mg/dL) than in July (26.9 ± 2.15 mg/dL) and December (27.8 ± 2.15 mg/dL; *p* < 0.05). Testosterone concentrations varied over time (*p* < 0.001), ranging from 1.18 to 5.96 ng/mL (mean: 3.21 ng/mL), and were higher in June (5.96 ± 0.82 ng/mL) than in July (1.76 ± 0.82 ng/mL), September (1.18 ± 0.82 ng/mL), November (1.44 ± 0.85 ng/mL) and December (1.49 ± 0.82 ng/mL; *p* < 0.05).

**Figure 4:**
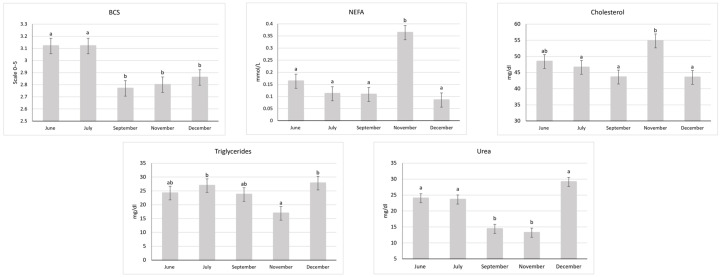
Mean (±SEM) body condition score (BCS) and circulating concentrations of NEFA, cholesterol, triglycerides, and urea in Sarda rams (*n* = 14) during the mating season. Differences in serially measured variables were analysed using linear mixed-effects models including time as a fixed effect and animal as a random effect to account for repeated measures. The effect of time was significant for BCS, NEFA, urea, cholesterol, and triglycerides (*p* < 0.001). Different letters above bars indicate statistically significant differences between time points (*p* < 0.05).

**Figure 5:**
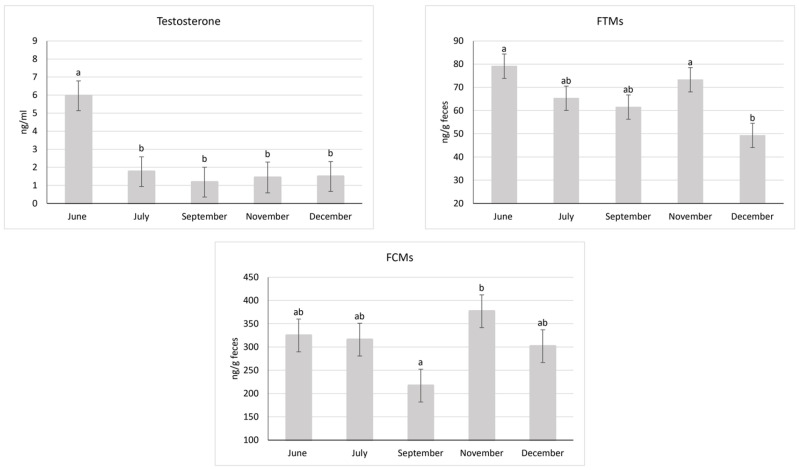
Mean (±SEM) circulating concentrations of testosterone, faecal thyroid hormone metabolites (FTMs) and faecal corticosteroid hormone metabolites (FCMs) in Sarda rams *(n* = 14) during the mating season. Differences in serially measured variables were analysed using linear mixed-effects models including time as a fixed effect and animal as a random effect to account for repeated measures. The effect of time was significant for testosterone and FTMs (*p* < 0.001) and for FCMs (*p* < 0.05). Different letters above the bars indicate statistically significant differences between months (*p* < 0.05).

FTMs varied during the mating season (*p* < 0.001), with a mean of 65.7 ng/g faeces (range: 48.6–77.1 ng/g) and were higher in June (77.1 ± 5.24 ng/g) and November (70.8 ± 5.24 ng/g) than in December (48.6 ± 5.24 ng/g; *p* < 0.05). FCMs also varied (*p* < 0.05), with a mean of 307 ng/g faeces (range: 183.9–354.3 ng/g) and were higher in November (354.3 ± 35.2 ng/g) than in September (183.9 ± 35.2 ng/g; *p* < 0.05). Overall variations in biochemical, hormonal, and body condition parameters during the mating season are shown in Figure 4 and Figure 5.

**Figure 6:**
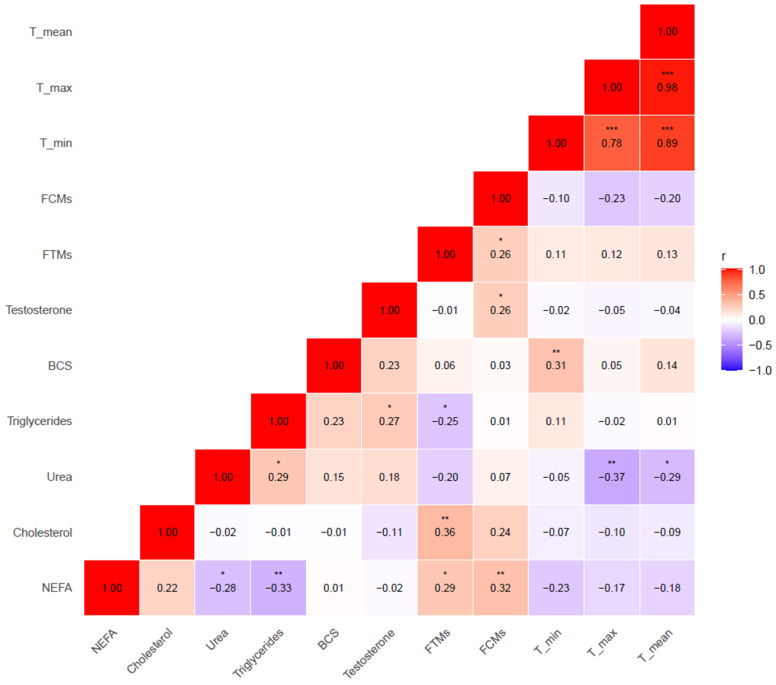
Correlation matrix showing the pairwise Pearson’s correlation coefficients (r) among all measured variables. The colour gradient indicates both the strength and direction of correlations: blue represents negative correlations, while red indicates positive ones. Asterisks indicate significance levels (* *p* < 0.05, ** *p* < 0.01, *** *p* < 0.001).

The results of correlation analyses among hormones, metabolic indicators and environmental temperatures are presented in a correlation heat map (Figure 6) reporting correlation coefficients (r) and their respective *p*-values. NEFA concentration was positively correlated with FTM (r = 0.29; *p* < 0.05) and FCMs (r = 0.32; *p* < 0.01) and was negatively correlated with urea (r = −0.28; *p* < 0.05) and triglycerides (r = −0.33; *p* < 0.01). Urea was negatively correlated with maximum (T-max; r = −0.37; *p* < 0.01) and mean temperature (T-mean; r = −0.29; *p* < 0.05). FTMs were positively correlated with cholesterol (r = 0.36; *p* < 0.01) and with NEFA (r = 0.29; *p* < 0.05). Testosterone was positively correlated with FCMs (r = 0.26; *p* < 0.05).

## 4. Discussion

This study evaluated seasonal fluctuations in metabolic parameters, key hormones, and body condition in Sarda rams (supplemented with exogenous melatonin approximately one month prior to breeding) during the mating season, together with flock reproductive outcomes assessed through ewe pregnancy status under commercial farming conditions.

The analysis of the reproductive outcomes revealed a dynamic breeding pattern, as shown by the higher number of conceptions registered between May/June and July/August for adult ewes and ewe lambs, respectively. These results can be interpreted in light of routine reproductive management in dairy ewes; the introduction of rams typically triggers the “ram effect,” increasing tonic LH secretion and inducing ovulation [34]. The resulting oestrus peaks reported in previous studies around 17–25 days after introduction [35] are consistent with the conception distribution observed in May and June in the present investigation. However, the reproductive workload evolved mid-season with the introduction of ewe lambs in late June. The lower PR observed in the intermediate checks can be potentially explained by their lower responsiveness to the ram effect in ewe lambs compared to adult ewes [2], likely contributing to the less synchronised and more dispersed breeding pattern observed between July and August. By the final checks (November and December), pregnancy rates returned to early-season levels, likely supported by a more favourable photoperiod.

Since pregnancy rate reflects the combined effects of ram activity, ewe fertility, and flock management, these findings should be interpreted within the context of the overall reproductive dynamics of the flock. In this context, PR was used as a descriptor of flock-level reproductive patterns, intended to support the interpretation of hormonal and metabolic patterns in rams, rather than to quantify individual ram fertility. This sustained breeding pattern, despite fluctuations in female availability, was accompanied by a significant decrease in BCS over time, suggesting mobilisation of body reserves in Sarda rams during the mating season. Notably, BCS remained above the critical threshold (BCS < 2.5) [19], suggesting that the energetic taxing did not reach a level capable of impairing overall success.

The metabolic profiles further elucidate these seasonal changes. NEFA concentrations remained low during the early months and rose significantly only in November. This trend suggests that while the rams were losing body condition throughout the season, they were not in a state of acute negative energy balance (NEB). The late-season spike in NEFA may reflect a combined effect of accumulated reproductive fatigue, decreased intake and poor-quality autumn pastures.

The associations observed between different parameters may reflect a coordinated metabolic response. A significant negative correlation between NEFA and triglycerides presumably reflects the limited hepatic capacity of ruminants to export triglycerides via VLDL [36,37]. Similarly, the lower urea levels observed between September and November may reflect changes in overall nitrogen balance and dietary protein utilisation during this high-exertion period. In response, urea synthesis may have been reduced to conserve energy and prioritise nitrogen retention [38]. The negative correlation between NEFA and urea, as well as the positive correlation between triglycerides and urea, suggests that these variables may be involved in the metabolic adaptations associated with prolonged energy requirements. Plasma urea levels were also negatively correlated with ambient temperature. In the literature, a positive correlation between circulating urea concentration and air temperatures is generally reported [39,40]; however, the negative correlation observed in the present investigation is consistent with the findings of Abou Akkada et al. [41], who suggested that both ambient temperature and nutritional factors contribute to the variation in plasma urea concentrations.

This metabolic adaptation was closely mirrored by hormonal shifts, particularly regarding the adrenal and thyroid axes. Cholesterol levels peaked in November, which may be attributed to both enhanced lipid mobilisation and serving as a precursor for the increased demand in steroidogenesis [42]. This interpretation is supported by the concomitant peak in FCMs, indicating heightened Hypothalamic–Pituitary–Adrenal (HPA) axis activity at the end of the season. During this period, the low number of ewes available for mating likely intensified reproductive competition, coinciding with inadequate intake. Moreover, FCMs were positively correlated with NEFA, suggesting a possible association between metabolic changes and allostatic load [43].

Interestingly, thyroid regulation appeared more closely associated with metabolic indicators than with environmental variables in this dataset. FTMs remained stable until a subtle modulation in December, despite cold temperatures that typically stimulate thermogenesis [44,45]. Adaptation to cold causes deiodination of thyroxine (T4) and thus promotes an increase in triiodothyronine (T3) levels in the blood in humans and animals [46]. In this study, nutritional and metabolic status, rather than ambient temperature, may have played a greater role in thyroid regulation in rams. This is consistent with patterns described in the literature [47], in which energy balance appears to be more relevant than environmental cues for long-term metabolic regulation. The positive correlation observed between FTMs and NEFA, and between FTMs and cholesterol, could suggest a metabolic adjustment in response to the high energy demands of the mating season. Unlike the metabolic stress response observed in animals under negative energy balance, where increased lipolysis is typically associated with a suppression of the hypothalamic-pituitary-thyroid (HPT) axis [47], our findings suggest that thyroid activity was not suppressed under the present conditions, and may be associated with lipid mobilisation and energy utilisation patterns [45]. This is consistent with evidence showing that thyroid hormones remain elevated when metabolic demands are high, provided the animal does not enter a critical state of energy deficit [47].

Finally, the gonadal axis showed a distinct temporal pattern in response to both reproductive management and endocrine stimulation. Testosterone levels showed an early peak in June, followed by a seasonal decline. The early peak may reflect the use of melatonin implants one month before the start of the mating season [48]. Moreover, close contact with oestrous ewes is known to rapidly elevate LH and testosterone in rams [49], thus it is plausible that such contact contributed to the high circulating testosterone concentrations measured in June. The subsequent stabilisation at lower levels, yet still sufficient to sustain reproductive activity in the rams [50], may be related to reduced sexual stimulation as the reproductive load decreased. Regardless, the endocrine profile observed in June should be interpreted within the context of a farm-managed, artificially advanced mating season rather than a purely natural photoperiodic pattern. In this context, the thyroid hormone profile should also be interpreted with caution, as melatonin treatment may interact with the photoperiodic regulation of the HPT axis and influence seasonal thyroid hormone signalling [51].

It is interesting to notice the positive correlation between testosterone and FCM observed in this study. It was expected that testosterone and FCMs levels would be negatively correlated, as the scientific literature supports the hypothesis of suppression of the hypothalamic pituitary gonadal axis when the HPA axis is activated [13,52]. Conversely, the positive correlation found between testosterone and cortisol is consistent with the findings of Wingfield et al. and Harden et al. [12,53], who described a positive association between the reproductive and stress axes, potentially reflecting a functional coupling of both systems during periods of increased energetic and reproductive demands. However, further studies are needed, as causality cannot be inferred from our results.

Taken together, these findings suggest that during the mating season in a commercial farm, Sarda rams exhibit changes in metabolic and hormonal profiles consistent with physiological adaptation to seasonal conditions. These patterns, observed alongside flock reproductive outcomes, provide a descriptive overview of seasonal physiological and reproductive dynamics in this production system.

This study has some limitations that should be acknowledged. First, reproductive outcomes were assessed at the flock level through pregnancy rate, which provides an indirect measure of reproductive performance and does not allow attribution of fertility outcomes to individual rams. Second, the study was conducted under commercial semi-extensive farming conditions, which ensured a real-world scenario, but limited full control over flock composition and environmental variability, as well as precluding the assessment of detailed reproductive parameters such as semen quality, rams’ libido and mating behaviour, which could have provided additional information on ram reproductive function.

## 5. Conclusions

In conclusion, this study describes temporal changes in metabolic parameters, key hormones and BCS in Sarda rams during the mating season, under semi-extensive field conditions. These changes, observed alongside stable flock reproductive outcomes, describe the physiological dynamics associated with prolonged reproductive activity in a commercial management system. While the reproductive period was associated with a progressive decline in body condition and a late season increase in lipolysis (NEFA), rams appear to maintain metabolic homeostasis without evidence of severe energy deficit. The positive correlation between testosterone and FCMs may be associated with the increased energetic and reproductive demands. Furthermore, the observed patterns of FTMs appeared to be more closely associated with indicators of energy balance than with environmental variables. These findings underscore the importance of monitoring BCS and metabolic markers during the final stages of the mating season (October–November), suggesting that this period may represent a strategic window for monitoring metabolic status and implementing nutritional management practices aimed at supporting ram performance under semi-extensive conditions.

## Data Availability

The original contributions presented in this study are included in the article. Further inquiries can be directed to the corresponding author.

## Supplementary Materials

The following supporting information can be downloaded at: https://www.mdpi.com/article/10.3390/ani16142196/s1, Table S1: Effect of age on metabolic, endocrine and body condition variables estimated using linear mixed models.
