# Supplementary material for: Metabolic, Hormonal and Body Condition Changes in Melatonin-Implanted Dairy Rams and Flock Pregnancy Rate During the Mating Season: A Longitudinal Field Study
Source: Animals (Basel). 2026 Jul 15;16(14):2196. doi: 10.3390/ani16142196 (PMC13405832; doi:10.3390/ani16142196)
Supplement: Supplementary file 1 [file animals-16-02196-s001.zip › animals-4331495-supplementary.pdf]

**Variable**	**Scale**	**NumDF**	**DenDF**	**F value**	**p-value**
NEFA	Original	1	64	3.730	0.058
Cholesterol	Original	1	12	0.134	0.720
Urea	Original	1	12	0.371	0.554
BCS	Original	1	12	0.005	0.943
Triglycerides	Log-transformed	1	12	0.171	0.687
Testosterone	Log-transformed	1	63	0.587	0.446
FTMs	Log-transformed	1	12	0.044	0.838
FCMs	Log-transformed	1	64	2.712	0.104

Table S1. Effect of age on metabolic, endocrine and body condition variables estimated using linear mixed models. No significant effect of age was detected for any variable ($p > 0.05$).
